# Retraction: Do surrounding people's emotions affect judgment of the central person's emotion? Comparing within cultural variation in holistic patterns of emotion perception in the multicultural Canadian society

**DOI:** 10.3389/fnhum.2024.1480750

**Published:** 2024-08-23

**Authors:** Frontiers Editorial Office

The journal retracts the 05 July 2022 article cited above.

Following publication, the authors contacted the Editorial Office to request the retraction of the cited article, stating that they had identified several critical errors in the reporting of the results from this study, conducted under the protocol approved by the University of Alberta Institutional Review Board (Pro00107588). These errors are as follows:

(i) Inaccurate reporting of the participant recruitment procedure,

(ii) Failure to disclose preceding research and data to the editor and the reviewers during the review process, and

(iii) Lack of proper attribution of credit to other researchers.

An investigation was conducted in accordance with Frontiers' policies that confirmed this; therefore, the article has been retracted. The authors agree to this retraction.

